# Schema therapy for Dissociative Identity Disorder: a case report

**DOI:** 10.3389/fpsyt.2023.1151872

**Published:** 2023-04-21

**Authors:** Nathan Bachrach, Marleen M. Rijkeboer, Arnoud Arntz, Rafaële J. C. Huntjens

**Affiliations:** ^1^Department of Medical and Clinical Psychology, Tilburg University, Tilburg, Netherlands; ^2^GGZ-Oost Brabant, Department of Personality Disorders, Helmond, Netherlands; ^3^Department of Clinical Psychological Science, Maastricht University, Maastricht, Netherlands; ^4^Department of Clinical Psychology, University of Amsterdam, Amsterdam, Netherlands; ^5^Department of Experimental Psychotherapy and Psychopathology, University of Groningen, Groningen, Netherlands

**Keywords:** schema therapy, Dissociative Identity Disorder, case report, PTSD, personality disorder

## Abstract

Treatment for Dissociative Identity Disorder (DID) often follows a practice-based psychodynamic psychotherapy approach that is conducted in three phases: symptom stabilization, trauma processing, and identity integration and rehabilitation. The percentage of patients that reach the third phase is relatively low, treatment duration is long, and the effects of this treatment on the core DID symptoms have been found to be small or absent, leaving room for improvement in the treatment of DID. Schema Therapy (ST) is an integrative psychotherapy that has been proposed as a treatment for DID. This approach is currently being investigated in several studies and has the potential to become an evidence-based treatment for DID. This case report presents an overview of the protocol adaptations for DID ST treatment. The presented case concerns a 43-year-old female patient with DID, depressive disorder (recurrent type), PTSD, cannabis use disorder, and BPD. Functioning was very low. She received 220 sessions of ST, which included direct trauma processing through Imagery Rescripting (ImRs). The patient improved in several domains: she experienced a reduction of PTSD symptoms, as well as dissociative symptoms, there were structural changes in the beliefs about the self, and loss of suicidal behaviors. After treatment she was able to stop her punitive mode, to express her feelings and needs to others, and to participate adequately in social interaction. This case report indicates that ST might be a viable treatment for DID, adding to a broader scope of treatment options for this patient group.

## Introduction

Dissociative Identity Disorder (DID) is a highly disabling disorder, associated with high levels of impairment, high risk for self-harm, multiple suicide attempts, high mortality, and very high societal costs (1). The main diagnostic criterion for DID is the perceived presence of two or more distinct identities, accompanied by a marked discontinuity in the sense of self and agency, and alterations in affect, behavior, consciousness, memory, perception, cognition, and/or sensory-motor functioning. Also, patients often report recurrent gaps in the recall of important personal information, everyday events, and traumatic events (2). The estimated 12-month prevalence of DID is 1.5% in the general American population (2), and around 5% in psychiatric settings (3).

Treatment for DID often follows a practice-based psychodynamic psychotherapy approach that is conducted in three phases: symptom stabilization, trauma processing, and identity integration and rehabilitation (4). The percentage of patients who reach the third phase of treatment is relatively low [17–33%, (5)] and treatment duration is long, on average 8.4 years (6). The effectiveness of this treatment has been examined in several non-controlled studies (6–8) and one Randomized Controlled Trial [RCT; (9)]. The results indicated that, although the general functioning of patients improved, the effects of this treatment on the core symptoms (i.e., dissociative symptoms) are small or absent. Hence, there is ample room for improvement in the treatment of DID.

Schema therapy (ST) has been introduced as a viable alternative treatment for DID (10–12). ST is thought to be applicable to and effective for DID for several reasons. First, ST as a whole, as well as its trauma processing component, Imagery Rescripting (ImRs), are effective for disorders that result from interpersonal trauma in childhood, including complex PTSD and personality disorders [e.g., (13–17)]. Secondly, ST was found to reduce dissociative symptoms in patients with Borderline Personality Disorder (BPD) (18). Thirdly, perceived shifts between identities in people with DID are understood as shifts between modes (temporary states of mind) and compartmentalization is not assumed (19). Extreme shifts in emotions, cognitions, and behaviors that are present in DID also appear in other disorders that are related to severe and prolonged childhood abuse, such as BPD; ST delivers tools for dealing with these shifts (20). Fourthly, a recent RCT (15) investigating the effectiveness of ImRs in people with PTSD as a result of early childhood trauma showed that trauma treatment is highly effective and can be performed safely without a stabilization phase. As a first illustration of this new approach to the treatment of DID, this case report presents an illustration of the application of an adapted form of ST for DID.

## Case description

Ella (fictitious name) is a 43-year-old patient with an extensive psychiatric history, who was referred to a specialized mental health center in the Netherlands to participate in a study on the treatment of DID with ST. Ella experienced nightmares and flashbacks about past traumatic experiences, and reported 17 identities, as well as dissociative amnesia (i.e., memory gaps for daily life events and traumas). Several identities were obsessed with self-hatred and self-punishment and repeatedly gave orders to hurt or kill herself. She broke her arm once by force, repeatedly cut herself on her arm, and attempted suicide several times. According to the patient's report, traumatic experiences involved recurrent sexual abuse by her father during her childhood (4–11 years), as well as several times by a teacher and a peer from secondary school. Her mother denied the abuse and behaved in a guilt-inducing way. Moreover, during her training as a dentist assistant after graduating from high school, a manager tried to sexually abuse her, after which she mentally broke down. She was hospitalized numerous times due to parasuicidal behavior and suicide attempts. She also received CBT for 3 years. This treatment focused on depressive and anxiety symptoms, (para)suicidal behaviors, and cannabis addiction. It was delivered in individual as well as group format and did not result in long-lasting results. Previous treatment in this case did not include trauma stabilization therapy. She met her husband 14 years ago and has a son who is 6 years old. She feels insecure about the upbringing of her son and feels unconnected to her partner. At the start of therapy, she was not able to work.

## Assessment

The patient gave informed consent for participation in the study and for the publication of this case report. The Structured Clinical Interview for DSM disorders Dissociative disorders-Revised [SCID-D-R; (21)], SCID-I, and SCID-II (22, 23) were used to assess the presence of clinical disorders by an independent experienced clinician. Ella was diagnosed with DID, depressive disorder, PTSD, cannabis use disorder, and BPD, and her Global Assessment of Functioning (2) score was 25. Table 1 shows the results of the baseline assessment. This case is part of a non-concurrent multiple baseline design study among 10 DID patients (10).

**Table 1:** Results of baseline measures.

**Measure**	**Baseline**
**SMI** **(****24** **)**	
Vulnerable child	58%
Angry child	46%
Enraged child	42%
Impulsive child	45%
Undisciplined child	52%
Happy child	24%
Compliant surrender	49%
Detached protector	47%
Self-soother	55%
Self-aggrandizer	36%
Bully and attack	27%
Punitive parent	80%
Demanding parent	74%
Healthy adult	40%
**SCID-D** (21)	
Amnesia	Severe
Derealisation	Severe
Depersonalization	Severe
Identity diffusion	Severe
Identity alteration	Severe
SCID-I (22)	
Depressive disorder	
Post-traumatic stress disorder	
Cannabis use disorder	
Global Assessment of Functioning	25
SCID-II (23).	
Borderline Personality Disorder	

## Treatment

The treatment consisted of 160 sessions twice per week, followed by 40 weekly sessions. Thereafter, she received 6 monthly booster sessions which were aimed at reconsolidation and generalization of ST insights and skills learned during the active treatment. Each session lasted 50 min. ST for DID follows the same theoretical framework and makes use of therapeutic interventions as originally developed by Young et al. (25), though ST for DID is personalized to each patient as they present with different symptoms. Furthermore, several important adaptations to ST were made to meet the needs of DID patients. These will now be discussed.

### Case-conceptualization and establishing a shared definition of problems in schema therapy language

**Figure 1:**
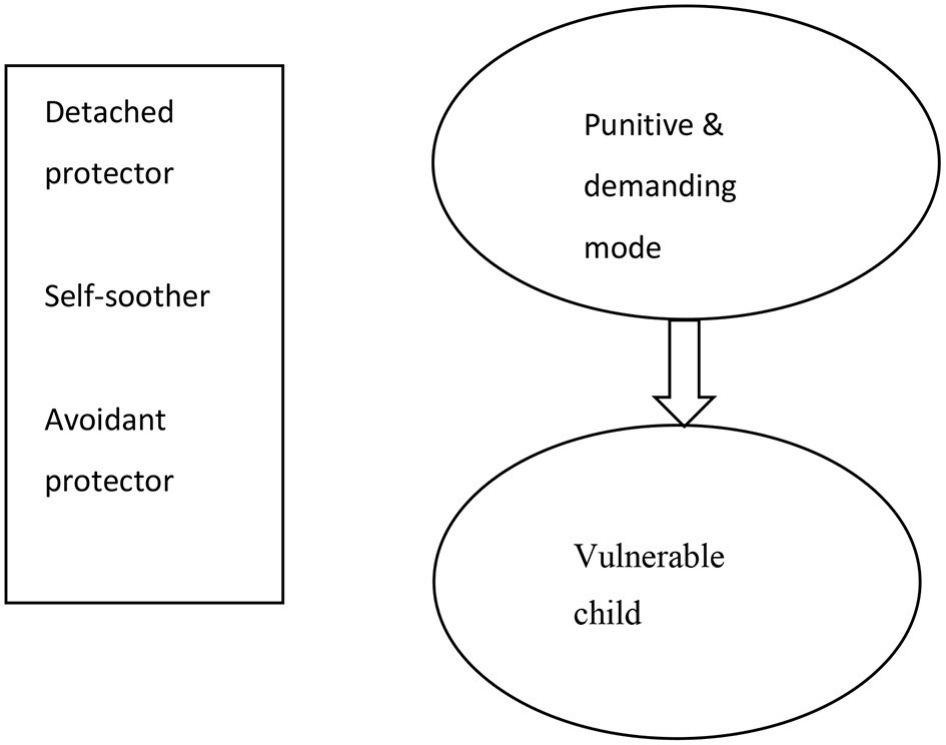
Schema mode model of the patient.

**Figure 2:**
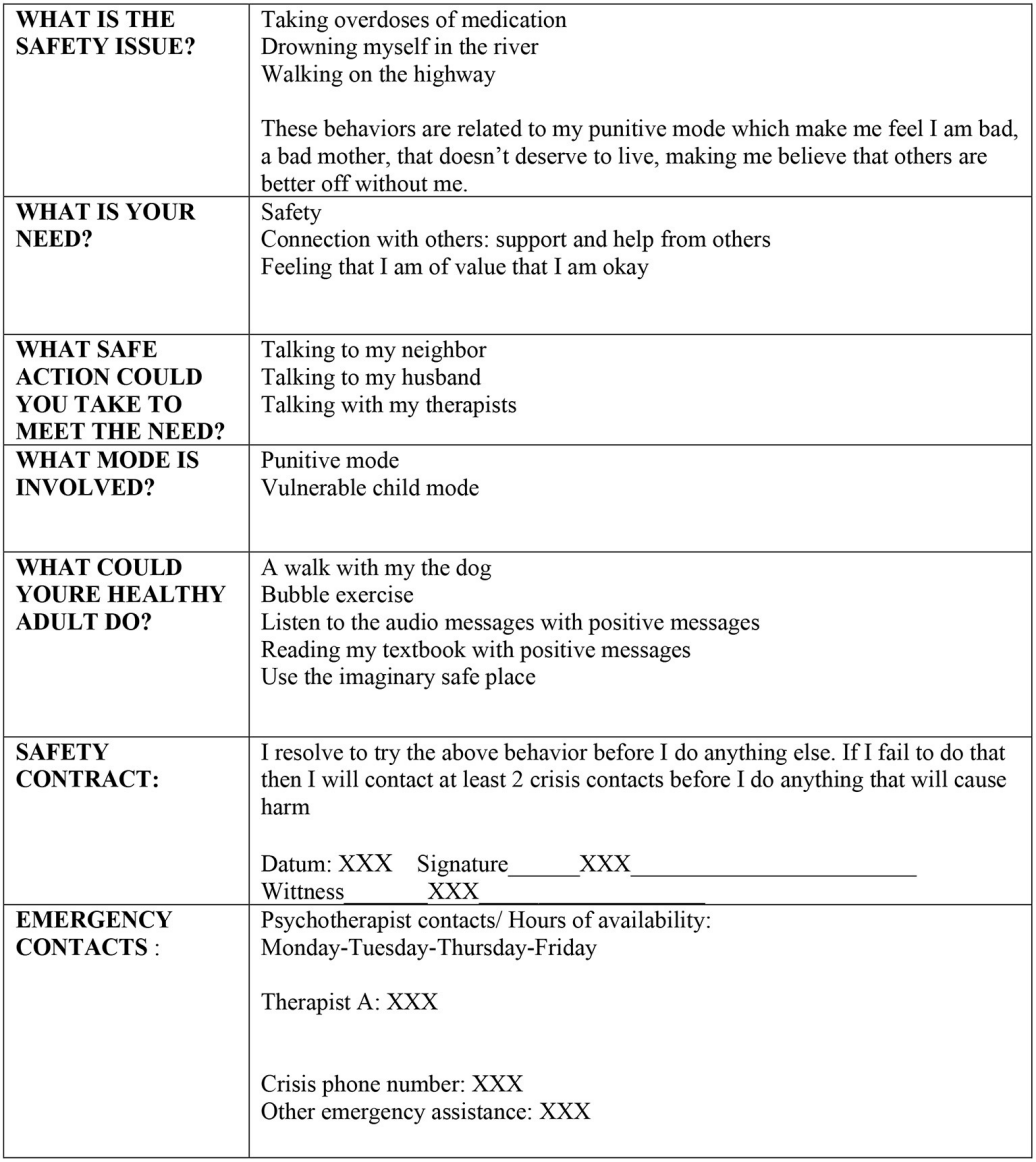
Safety plan of the patient.

At the start of treatment, the diagnosis of DID as well as the main problems of the patient were discussed. Ella was educated on the rationale of ST for DID with regards to basic needs and how frustration of these needs leads to schemas, modes, and psychopathology. To manage expectations, conditions of treatment were explained, such as treatment length (3.5 years), frequency of sessions, need for active participation, whom to contact in case of crisis, and the availability of the therapist. Much effort was put into building a working alliance throughout treatment by validating thoughts and emotions and being present, available, and consistent. Being really determined in finding solutions to deal with severe and persistent symptoms, not giving up but instead delivering hope and power is very important in working with DID patients. She was educated on how DID is understood in terms of schema theory (as modes), and identity states were thereafter translated into modes by clustering identities by their function and reformulating and merging them into a mode (see Table 2). There was no pressure to share all identities; the therapist worked with states that were present. Together with Ella a mode model was made (see Figure 1), containing the most prevalent modes: punitive and demanding mode (e.g., internal demanding and punitive messages), the vulnerable child mode (painful feelings, PTSD symptoms), the detached protector (e.g., withdrawing and disconnecting), avoidant protector (active avoidance behaviors), and self-soother (using cannabis and auto-mutilation to deal with painful feelings). This idiosyncratic model was consistent with the results of a recent empirical study into the most prevalent modes in patients suffering from DID (26). Moreover, (para)suicidal behaviors, coping mechanisms, and supportive relatives were assessed (level of parasuicidal behaviors was high and healthy coping mechanisms low), after which a basic safety plan was made in which Ella agreed to try to perform helpful behaviors (e.g., talking to my neighbor, talking to my husband, talking with my therapists) before harming herself (see Figure 2). This plan was used whenever basic safety became an issue, evident for example by the patient sending an appeal for help by e-mail or phone. She e-mailed texts like “*Death must be met with dignity. It is the only dignified thing left to do. I am never going to recover and if you really get to know me you would see how bad we are. I don't deserve to live*.” Yet, it was possible to reassure her and prevent self-harm through email and short phone calls.

**Table 2:** Overview of parts and the corresponding modes.

	**Name of identity state**	**Age**	**Behavior**	**Function of the behavior = what is it trying to achieve or prevent?**	**What triggers the identity state?**	**Feelings**	**Needs**	**Mode**
1	A	40	Presenting myself as a strong and responsible wife	Making sure things go well in here and now, prevent panic and chaos in here and now prevent clutter	Clutter Anxiety	Lack of feelings	Safety	Detached protector
2	M	43	Presenting myself as tough, with courage arrogant, independent	Being in control	Anxiety People who come close	Lack of feelings	Safety	Detached protector
3	L	35	Restless behavior	Denies traumas, believes that we are evil because we made up everything	Anything that has to do with justice or jurisprudence	Anxiety and anger	Safety and sense of identity	Detached protector Punitive mode
4	S	46	Faithful, puts everything in God's hands	Letting go of responsibilities	Churches crosses everything regarding faith	Lack of feelings	Autonomy, competence, and sense of identity	Compliant surrender
5	E	48	Striving, doing my best at everything	Wants compliments, tries to avoid disapproval	Deadlines appointments	Anxious	Autonomy, competence, and sense of identity	Perfectionistic over-controller
6	N	25	Avoid difficult situations	Preventing rejection/failure	Social situations Situations in which I need to perform	Anxiety	Autonomy, competence, and sense of identity	Avoidant protector
7	F	14	Getting out of social situations	Preventing rejection/failure	Social situations Situations in which I need to perform tasks	Anxiety	Autonomy, competence, and sense of identity	Avoidant protector
8	M	No age	Being inconspicuous, behaviors to avoid criticism.	Trying to prevent mistakes, avoid punishment.	Loud men's voices, anger	Lack of feelings	Safety	Detached protector
9	L	No age	Destructive behavior seeks danger, wanders at night	Punishing myself	Crying or anxious children	Anger	Safety and nurturance autonomy, competence, and sense of identity	Punitive mode
10	C	25	Avoiding traumatic memories by distraction	Avoiding pain	Triggers for traumas	Lack of feelings	Safety and nurturance autonomy, competence, and sense of identity	Detached protector
11	M	47	Arrogant, charming strong punishes others	Feeling in control, reduction of anxiety	Patronizing behavior of others, submissiveness of myself	Feeling strong	Safety and nurturance	Bully and attack
12	M	51	Waiting, subservient and quiet, following submissively	Trying to make things up again	Arguing, loud voices	Anxiety	Safety and nurturance	Compliant surrender
13	P	7	Scared	Afraid of men and loud voices, afraid of abuse.	Men	Fear	Safety and nurturance	Vulnerable child
14	L	No age	Cleaning up, keeping everything neat, sorting	Trying to prevent critic	Clutter, toys and anger from others, conflict	Fear	Safety and nurturance	Perfectionistic over-controller
15	S	5	Anxious and small, crawling away, thumb in mouth	Fear of abuse and violence	Men	Fear	Safety and nurturance	Vulnerable child
16	J	No age	Takes care for the little ones	Takes care for the little ones	The little ones	Warm feeling	Safety and protection Autonomy, competence, and sense of identity	Healthy adult
17	K	No age	Running away from home, seeks safety	Reduction of anxiety	Quarrel	Anxiety	Safety and protection	Avoidant protector

### Dealing with dissociation and working with the detached protector

Specific adaptations in ST were made to address dissociative responses. Ella was educated on dissociation, stressing that it is a natural reaction to extreme and ongoing stress, especially when (biologically) sensitive to stressors. Furthermore, dissociative behaviors such as detachment or being unresponsive to stimuli from the environment were framed as behaviors that once had a clear survival function, but at present were mainly maladaptive. A strip of fleece was used to make a literal connection between Ella and the therapist, and to gain control over what was happening during the session. Whenever Ella zoned out or started to dissociate, the therapist gave the fleece a slight tug to have her stay connected and more present. Also, Ella could tug the fleece whenever she was in need, e.g., when the pace of the therapist was too high. At the beginning, the tugging and exploration of what triggered the disconnection was mainly initiated by the therapist, but gradually Ella became more active in tugging and exploring. Other techniques that were used to stop disconnection were grounding, such as the “Stop, Freeze, and Breathe” exercise (27), naming five things you see, throwing a small ball, or pinching some things hard (a shell or a sharp wooden stick). Also, the therapist and Ella found out that a dog clicker helped Ella to orient in the present whenever she got overwhelmed by flashbacks. She used the clicker when she sensed that she was (about to) reexperience traumatic events. The clicker helped her to feel in control over flashbacks and reorient to the present. Moreover, chair exercises, such as interviews with the detached protector, validating its protective function in the past, asking it to be less present, and setting the chair more aside in order to connect and reparent the vulnerable child, were used to reduce detachment.

### Working with the avoidant protector

Avoidance behaviors are highly prevalent in DID patients and are a strong maintaining factor. Therefore, in ST for DID there is a constant alertness for avoidance behavior shown by the various identities. Dependent on their function they are reframed as a coping mode. Because the avoidance behavior can be intense and strong, creative solutions on how to deal with it are needed.

Ella had a strong avoidant protector (interpersonal and situational avoidance). She tended to avoid multiple situations (e.g., talking to other mothers at the schoolyard, attending other social situations, or discussing shameful past situations with the therapist). Her awareness of avoidance increased by teaching her to identify the behaviors of the avoidant protector and turn her attention toward avoidance behaviors in and outside the sessions via homework assignments (mode awareness work sheets). Avoidance patterns were targeted by chair work [dialogue with the avoidant protector, validation of the protective function in the past, asking the mode to make space for healing of the vulnerable part, and empathic confrontation (e.g., confronting her with the fact that avoiding trauma processing maintains PTSD, and not going along with avoidance)]. Creative solutions were used to break through her avoidance (e.g., picking her up from the parking lot and outside the building when she was afraid to enter the health center building and using telehealth when she wanted to cancel a therapy session combined with discussing her avoidance). In addition to cognitive interventions such as exploring the pros and cons of avoidance, she was stimulated to exercise approach behaviors at home (e.g., sharing feelings with partner or talking to other moms). Gradually, Ella became more able to diminish her tendency to avoid in therapy, as well as in daily life situations.

### Working with the self-soother

DID patients frequently use alcohol, drugs, or medication to avoid dealing with intense negative emotions. In ST for DID these behaviors are reframed as the self-soother mode. The patient is made responsible for her behavior instead of going along with her tendency to attribute her behavior to an identity over which she has no control.

In the case of Ella, her cannabis use was framed as an avoidance strategy; she used cannabis daily to avoid painful feelings from past traumatic experiences. After several attempts to reach abstinence of cannabis through CBT techniques for addiction used in the context of ST, an additional clinical detox at her request helped Ella to stop her cannabis use completely. During this detox she expressed that she did not get overwhelmed by flashbacks and painful feelings, which helped her to continue abstinence, because sedation was not necessary anymore.

### Trauma processing

In ST for DID, trauma processing is seen as a crucial part of therapy, which needs to start as soon as possible (usually several weeks to a few months). In ST for DID there is no stabilization phase in which skill and emotion regulation strategies are taught nor is stabilization of symptoms a prerequisite for trauma processing, whilst trauma processing in itself is found to have a stabilizing effect in patients suffering from severe childhood traumas [e.g., (17)]. Trauma processing is done by ImRs, a technique that aims to change the dysfunctional meaning of early aversive experiences. It consists of prompting patients to rescript painful autobiographical memories in line with their unmet needs (28). To adapt ST to the specific needs of DID patients, the use of ImRs has been broken down in steps, to customize the pace of trauma processing and level of trauma exposure to what patients are able to deal with, gradually increasing the level of exposure and the involvement of the healthy adult part of the patient. In the case of Ella, trauma processing started after 8 weeks. This was possible due to several factors such as raising her commitment, the good working alliance, not avoiding trauma work but carrying it out at a level that was manageable for her, performing it in small steps, and the high frequency of treatment sessions. Imagery work was built up slowly, starting with a neutral experience (imagining skiing together with the therapist), whereafter mild negative (soothing of her crying as a child or being excluded at school) and more adverse negative experiences were processed (neglect and abuse experiences by father, teacher, and peer). ImRs was performed in small steps in which first the therapist rescripted, whereafter Ella was motivated to gradually participate in the rescripting (“*what would you like to say to him, okay just say that*”), and finally carrying out the rescripting herself. In the first 2 years, trauma work often disrupted her, because it activated the punitive part, sometimes leading to (para)suicidal behaviors. Therefore, in ST for DID one frequently oscillates between trauma work and working with the punitive part. At these moments, the safety plan was used and if necessary we worked with the punitive mode in the next session. In Ella, the punitive part told her she was bad and faulty and it was not worth living, making it very difficult to take care of the needs of the vulnerable child. The therapist interspersed ImRs with punitive mode work (see next paragraph) and stimulating adult healthy perspectives on feelings and needs of people. At the start the therapist kept the trauma work short (5 min) and gradually increased the duration of trauma processing (to about 30–40 min in one session). Over time, Ella thus increasingly tolerated trauma work and gained power over the traumatic experiences.

It took a long time and many repetitions before she was able to comfort and fulfill the needs of her vulnerable child. Only in the 3rd year she was able to adopt a healthier perspective on who was guilty and responsible for the abuse. In the last year she was able to rescript on her own. As a tool for performing the rescripting at home, she made a collage for each individual person who abused her to visualize the rescripting. It contained pictures of actions to stop the abuser (hitting him with a baseball bat, stabbing him with a knife, or setting fire to the house/school where the abuse took place). Additionally, it contained messages to say to the abuser (*shame on you, you're bad*), actions to bring the vulnerable child to safety (bring her to the hospital, wrapping her in warm blankets, or bringing her to a new safe home), and sentences to emphasize the innocence of the child and to build her self-worth (“*it is not your fault, there is nothing wrong with you*”).

### Banishing the punitive part

In ST for DID, aggressive, punitive, and highly demanding identities are reframed as the punitive and demanding mode. Repeated, persistent, and creative ways of fighting their messages and banishment are needed to reduce the impact on the patient. ST aims to stop these messages and to increase control over them by replacing them with realistic, healthier messages.

In Ella the punitive and demanding modes (e.g., telling her she was bad, guilty, worthless, and incapable) were highly prevalent and persistent, and had a profound impact on her quality of life. They played an important role in eliciting and maintaining strong negative feelings and thoughts, self-harm (e.g., damaging her arm), and suicide attempts (by auto-intoxication). In those moments, the safety plan was initially used, followed by punitive mode work. Early in therapy, Ella felt that getting rid of the punitive mode was invalidating, because she felt that it was a part of her, and she was afraid of losing other identities as well. Repeated education and exploration of the impact of the punitive and demanding modes was necessary to work on banishing the punitive and demanding modes. Through time, and after numerous repetition of these exercises, the impact of the punitive mode was diminished. Numerous ST techniques were used in this process, such as balloon techniques (e.g., putting an imaginative protective balloon around herself to shield her from the negative messages and blowing punitive messages into a balloon after which the balloon was released). Other techniques used were imaginative muting of the mode (using a remote control to diminish the volume or using duct tape to silence the voice), shrinking the punitive mode to a smaller size, incarcerating it, chair work (e.g., putting the punitive mode on a chair, ordering it to stop, and placing it outside the room), and rituals such as burying and burning the images and messages of the punitive mode. A major breakthrough was achieved during a clinical admission due to a suicide attempt induced by the punitive mode. At this moment in time, the therapist had become really fed up with the punitive mode, and authentically and very strongly directly addressed this mode: “*I want you to get out of Ella's life, you are making her life miserable. You must leave*.” Thereafter, the therapist motivated Ella to take back control and to bid farewell to the punitive mode once and for all. During an imagery exercise that followed, she imagined the punitive mode to change into a statue whereafter she shrank it, and chopped it into a thousand pieces. In the sessions that followed, Ella reported that the punitive part did not return, but she felt an empty hole within herself. The therapist and patient filled this hole with helpful messages for her vulnerable child.

### Healing the vulnerable child mode

In ST for DID, child identities are conceptualized as vulnerable child modes. The therapist frequently and repeatedly reparents the vulnerable child, using imagination exercises to fulfill the needs of the vulnerable child, and gradually stimulating the healthy adult part of the patient to participate in healing the vulnerable child. Ella did not show her vulnerable side during the first treatment sessions. She feared maltreatment by the therapist. It was possible to gain her trust by creating a sense of safety within the therapy, after which she was able to let the therapist get in contact with the vulnerable child. The high treatment frequency, repeated validation of feelings and needs, and availability of the therapist might have all contributed to the relatively quick formation of a good working alliance. The therapist reparented the vulnerable child by validating and comforting Ella, but also by educating her on universal basic rights and needs of children, and responsibilities of parents as well as by recurrent rescripting of traumatic events that contributed to her negative self-image and guilt and shame feelings.

### Stimulating autonomy

In ST for DID there is a strong focus on the stimulation of autonomy and taking responsibility for changing lifelong patterns throughout the treatment, because of the high levels of learned helplessness in DID patients. Ella often felt overwhelmed by her symptoms and unable to cope with most aspects of her life. Right at the start of treatment, personal goals were formulated to increase commitment and take responsibility for direction of the treatment. Also, homework exercises were given, in which Ella was asked to make summaries of each session, and was stimulated to express feelings and needs within sessions and at home (“*What does your little child mode think, feel, and need, and what does your healthy adult mode want to say to your father?*”). Especially in the last year of therapy, instead of doing the work for her, the therapist stimulated Ella to become more personally active in interventions. Autonomy and mastery were also stimulated by building a clear identity, figuring out what her likes and dislikes were, and which societal goals she wanted to pursue. In the last few months of treatment, the therapist and patient made a mode management plan together, in which all the helping interventions were included.

### Review of successes

Because of a persistence of symptoms and strong feelings of helplessness, continuous focusing on the strengths of the patients and the progress they make is very important. Every 6 months, successes were reviewed by both the therapist and Ella by looking back at the positive steps she made (e.g., “*You completely stopped using cannabis for 6 months now*”, “*Lately, you were able to stay present during each entire session*”, and “*You were able to rescript yourself* ”), and by looking at changes in the Mode Pie Chart [a pie chart in which the relative attendance of each mode is estimated; see (27)].

## Discussion

The effectiveness of ST for DID is currently being investigated in two non-concurrent multiple baseline design studies in the Netherlands (10). This case report is one of the first descriptions of the practical application of ST for DID [also see (12, 29)], and illustrates that ST might be a viable and effective treatment for DID. Ella reported dissociative amnesia for traumatic experiences at the start of treatment. However, during therapy she shared that she was able to access traumatic experiences but feared confrontation and thus tried to avoid them. ImRs helped her to gradually process these traumas. ImRs was adapted to the limitations of Ella; it started as soon as possible (after several weeks), was built up gradually, and was performed continuously during the course of treatment. Furthermore, she was able to go along with a new conceptualization of the self in terms of modes instead of identities.

Ella showed strong improvement in psychiatric symptoms; there was a strong reduction of dissociative symptoms, PTSD, and depression symptoms including absence of suicidal behaviors, and abstinence from cannabis. She improved in social interaction and societal participation: she now takes care of her son and dog, her relationship with her husband has improved, she is meeting with friends, and sings in a choir. She also works as a volunteer for a needy elderly person and is applying for a job as a dentist assistant. These results are in line with studies into the effectiveness of ST and ImRs in adjacent populations (17, 30). Ella found the termination of treatment very difficult, especially saying farewell to her therapist. Working so closely together during several years created a strong attachment bond, and ending of treatment can be difficult for both therapist and patient. Furthermore, because of the descriptive nature of this case report, no conclusion can be drawn about the evidence base of ST for DID; follow-up assessments were performed but cannot be presented because this case is part of a non-concurrent multiple baseline design study amongst 10 DID patients which is not yet finalized, so the results of individual participants cannot be shared (10).

## Conclusion

This case report shows how ST can be applied to DID and suggests the possible effectiveness of ST for DID in general. An important next step is to systematically investigate the effectiveness of ST for DID in methodologically well-designed treatment studies, possibly leading to evidence-based treatments that go beyond stabilization of symptoms.

## Patient perspective

Ella reported that ST for DID was and still is hard work. She has learned tools with which she can take and keep more control over modes and flashbacks. Where she used to avoid many situations and places, she now has the confidence to know that she can manage these on her own.

## Data availability statement

The original contributions presented in the study are included in the article/supplementary material, further inquiries can be directed to the corresponding author.

## Ethics statement

The studies involving human participants were reviewed and approved by the Ethics Committee of the Faculty of Behavioral and Social Sciences of the University of Groningen (EC-GMW). The patients/participants provided their written informed consent to participate in this study. Written informed consent was obtained from the individual(s) for the publication of any potentially identifiable images or data included in this article.

## Author contributions

NB wrote the first draft of the manuscript. All authors read, commented on, and approved the manuscript.
